# CNApy: a CellNetAnalyzer GUI in Python for analyzing and designing metabolic networks

**DOI:** 10.1093/bioinformatics/btab828

**Published:** 2021-12-08

**Authors:** Sven Thiele, Axel von Kamp, Pavlos Stephanos Bekiaris, Philipp Schneider, Steffen Klamt

**Affiliations:** Analysis and Redesign of Biological Networks, Max Planck Institute for Dynamics of Complex Technical Systems, Magdeburg 39106, Germany; Analysis and Redesign of Biological Networks, Max Planck Institute for Dynamics of Complex Technical Systems, Magdeburg 39106, Germany; Analysis and Redesign of Biological Networks, Max Planck Institute for Dynamics of Complex Technical Systems, Magdeburg 39106, Germany; Analysis and Redesign of Biological Networks, Max Planck Institute for Dynamics of Complex Technical Systems, Magdeburg 39106, Germany; Analysis and Redesign of Biological Networks, Max Planck Institute for Dynamics of Complex Technical Systems, Magdeburg 39106, Germany

## Abstract

**Summary:**

Constraint-based reconstruction and analysis (COBRA) is a widely used modeling framework for analyzing and designing metabolic networks. Here, we present CNApy, an open-source cross-platform desktop application written in Python, which offers a state-of-the-art graphical front-end for the intuitive analysis of metabolic networks with COBRA methods. While the basic look-and-feel of CNApy is similar to the user interface of the MATLAB toolbox CellNetAnalyzer, it provides various enhanced features by using components of the powerful Qt library. CNApy supports a number of standard and advanced COBRA techniques and further functionalities can be easily embedded in its GUI facilitating modular extension in the future.

**Availability and implementation:**

CNApy can be installed via conda and its source code is freely available at https://github.com/cnapy-org/CNApy under the Apache 2 license.

## 1 Introduction

Constraint-based reconstruction and analysis (COBRA) has become a powerful and widely used modeling framework for analyzing and redesigning metabolic networks (Bordbar *et al.*, 2014). Several software packages in different environments have been developed to support COBRA studies. These include command line-based tools, such as the MATLAB-based COBRA toolbox (Heirendt *et al.*, 2019) or the Python packages COBRApy (Ebrahim *et al.*, 2013) and ReFramed (https://github.com/cdanielmachado/reframed), as well as software with graphical user interface (GUI), e.g. OptFlux (Rocha *et al.*, 2010), implemented in Java, or the web-based platform DD-DeCaf (http://dd-decaf.eu/). Various constraint-based analysis techniques are also provided by the MATLAB toolbox *CellNetAnalyzer* (CNA) (Klamt *et al.*, 2007; von Kamp *et al.*, 2017), where these methods can be accessed within a GUI (via interactive network maps) or from command line (via API functions). The interactive network maps are a characteristic feature of CNA and support typical use cases of COBRA-based analyses within network visualizations. An example of such a use case is a data input (e.g. measured metabolic fluxes), followed by a computation (e.g. growth rate maximization) and the display of the resulting metabolic flux distribution in the maps.

To overcome the dependency on proprietary software and to enable the integration of more advanced GUI features, we developed the Python package CNApy, which is presented herein. CNApy adopts CNA’s basic concept of interactive network maps but extends it with various enhanced features for an interactive analysis of COBRA models and allows the connection to the universe of Python-based packages and modules.

## 2 Implementation and features

### 2.1 Architecture of CNApy

CNApy is a cross-platform desktop application written in Python that provides a state-of-the-art graphical front-end for the intuitive analysis of metabolic models with COBRA methods. The metabolic model is internally part of an instance of a CNApy project class, which also contains metadata, e.g. the associated network maps. The metabolic model can be built from scratch within the GUI or be imported from a Systems Biology Markup Language (SBML) file (Keating *et al.*, 2020). Import/export of metabolic models in SBML format uses functionalities of the COBRApy package (Ebrahim *et al.*, 2013) and the models are internally represented as COBRApy model objects. This also allows the direct use of standard COBRA analysis methods [such as flux balance analysis (FBA), flux variability analysis (FVA) etc.] provided by COBRApy. Further analyses, such as computation of elementary flux modes (EFM) and elementary flux vectors (EFV) (Klamt *et al.*, 2017) or of minimal cut sets (MCS) (Schneider *et al.*, 2020), are directly supported in CNApy by newly developed custom stand-alone Python packages (these can also be found on https://github.com/cnapy-org). Certain advanced features of EFM, EFV and MCS computation currently require functions of the original CNA toolbox. These functions can be accessed (on the fly) through a MATLAB or, as an open-source alternative, Octave bridge. The results of these computations are reported to CNApy, where the project is updated and results are displayed in the user interface. The same approach is currently also used for yield optimization (which is more complicated than classical FBA) (Klamt *et al.*, 2018). The MATLAB/Octave bridge could also be employed to perform specific calculations with other packages, such as the COBRA toolbox. However, outsourcing of advanced calculations will become obsolete when modules for the respective algorithms become available in Python.

Regarding the graphical front-end, CNApy implements a Model–View–Controller (MVC) architecture. The MVC model is realized in the form of an application state class. This class includes the data of the currently loaded project as well as data for the general program settings and user interface features. The view and the controller make use of objects (widgets) of the powerful Qt library (https://www.qt.io/). These objects include maps, lists, diagrams etc. and are integrated in a single application window. The advanced cross-platform UI toolkit Qt is accessed by its Pyside2 binding for Python (https://pypi.org/project/PySide2/). In contrast to CNA written in MATLAB, Qt allows the use of network graphics in scalable vector graphics format enabling an improved visualization of (and zooming in) metabolic network maps. As in CNA, the metabolic maps must be provided or generated by the user, e.g. by using available pathway maps from web resources (such as the BioCyc or KEGG database) or by drawing metabolic maps using general drawing programs (e.g. Inkscape) or specialized tools for metabolic networks, such as Escher (King *et al.*, 2015) or OMIX (Droste *et al.*, 2013). However, GUI-based model analysis is also possible and useful without any network visualization.

### 2.2 Key features of CNApy

**Fig. 1.**
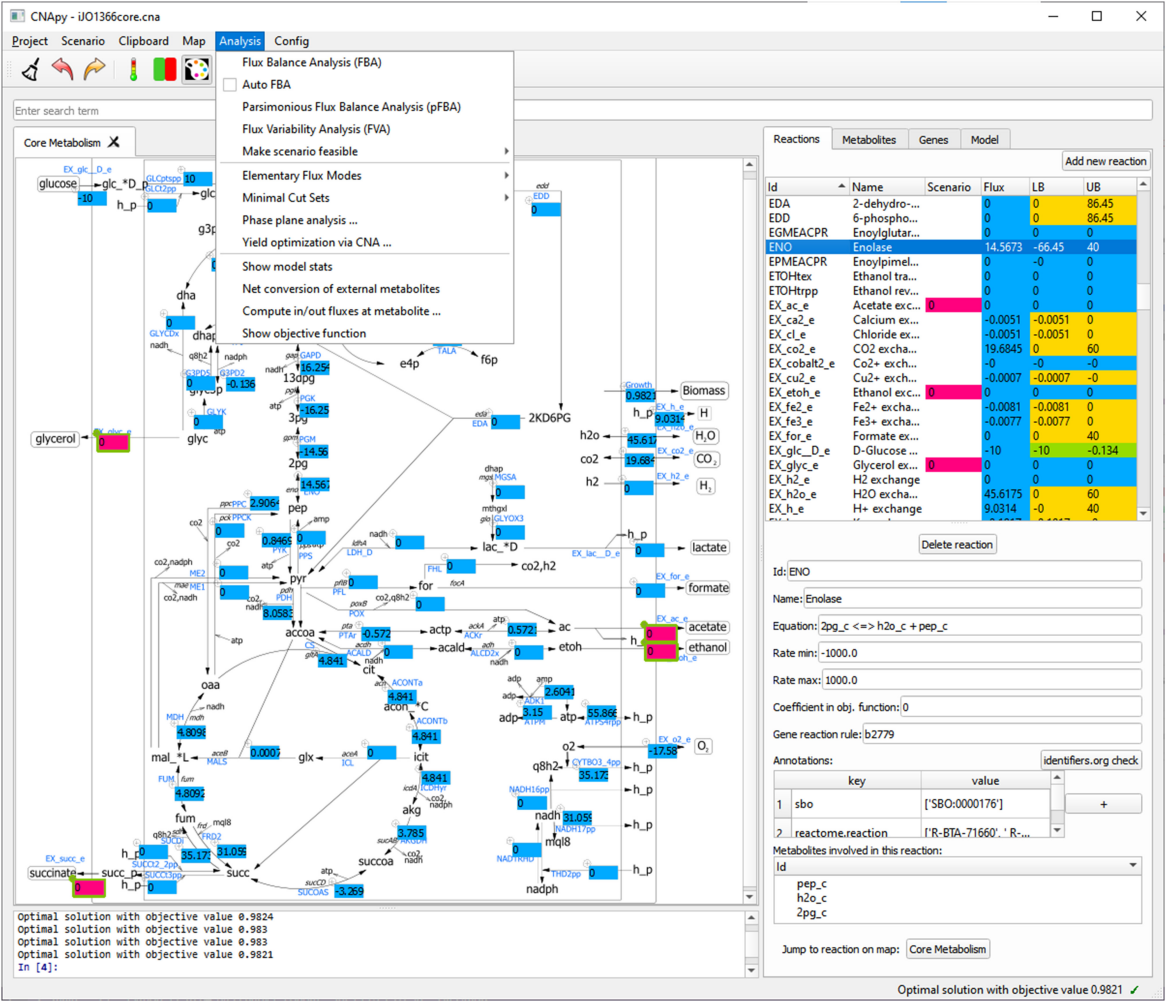
Screenshot of CNApy

CNApy supports metabolic network analysis with various standard and advanced COBRA methods including FBA, FVA, parsimonious FBA, phase plane analysis, yield optimization and computation of EFM, EFV and MCS (as mentioned above, partially via the MATLAB/Octave engine connecting CNApy with CNA). While many improvements over the original CNA arise from the usage of a modern UI toolkit, CNApy also offers smoother workflows. For example, the setup, import/export and editing of flux scenarios has been simplified and an edit history allows one to undo/redo changes in a scenario. CNApy projects can now be saved in a self-contained project file (*.cna) that includes graphics, the metabolic (SBML) model and other metadata. This makes it easier to copy and share CNApy projects. Additionally, it is now possible to import and export the coordinates of reaction boxes in a map, which allows reuse of maps and text coordinates in different projects. Further, CNApy facilitates an intuitive exploration and traversing of the network model, e.g. by jumping from a reaction to its associated metabolites and from a metabolite to its associated reactions. With this feature, and since calculation results (fluxes) are also displayed in the reaction list (see Fig. 1, right side), efficient model inspection and analysis is possible even if a network visualization is not available. Finally, CNApy integrates a Jupyter python console that allows the user to directly interact with the application from command line. Several example projects of CNApy (including genome-scale and core models of *Escherichia* *coli*) are provided in a project repository at https://github.com/cnapy-org/CNApy-projects.

## 3 Conclusion

CNApy is a new stand-alone desktop application with a powerful and user-friendly graphical front-end for metabolic network analysis and it is the first of its kind developed in Python. CNApy provides several unique GUI features including integrated model navigation and editing. It already supports a number of standard and advanced COBRA techniques, but other routines and new algorithms can easily be embedded in its GUI facilitating modular extension of the toolbox in the future. CNApy is an open-source project and contributions are encouraged on GitHub (https://github.com/cnapy-org).

## Funding

This work was supported by the German Federal Ministry of Education and Research (de.NBI partner project ‘ModSim’ (FKZ: 031L104B)]; and by the European Research Council (721176).

*Conflict of Interest*: none declared. 
